# Utilizing Molecular Dynamics Simulations, Machine Learning, Cryo-EM, and NMR Spectroscopy to Predict and Validate Protein Dynamics

**DOI:** 10.3390/ijms25179725

**Published:** 2024-09-08

**Authors:** Ahrum Son, Woojin Kim, Jongham Park, Wonseok Lee, Yerim Lee, Seongyun Choi, Hyunsoo Kim

**Affiliations:** 1 Department of Molecular Medicine, Scripps Research, San Diego, CA 92037, USA; ahson@scripps.edu (A.S.); 2 Department of Bio-AI Convergence, Chungnam National University, 99 Daehak-ro, Yuseong-gu, Daejeon 34134, Republic of Korea; woojin1544@gmail.com (W.K.); 975pjh@gmail.com (J.P.); wonseogi46@gmail.com (W.L.); leeyarim53@gmail.com (Y.L.); 3 Department of Convergent Bioscience and Informatics, Chungnam National University, 99 Daehak-ro, Yuseong-gu, Daejeon 34134, Republic of Korea; akfhd1212@gmail.com (S.C.); 4 Protein AI Design Institute, Chungnam National University, 99 Daehak-ro, Yuseong-gu, Daejeon 34134, Republic of Korea; 5 SCICS, 99 Daehak-ro, Yuseong-gu, Daejeon 34134, Republic of Korea

**Keywords:** protein dynamics, structural biology, molecular dynamics, allosteric regulation, enzyme catalysis, artificial intelligence, protein folding, conformational changes

## Abstract

Protein dynamics play a crucial role in biological function, encompassing motions ranging from atomic vibrations to large-scale conformational changes. Recent advancements in experimental techniques, computational methods, and artificial intelligence have revolutionized our understanding of protein dynamics. Nuclear magnetic resonance spectroscopy provides atomic-resolution insights, while molecular dynamics simulations offer detailed trajectories of protein motions. Computational methods applied to X-ray crystallography and cryo-electron microscopy (cryo-EM) have enabled the exploration of protein dynamics, capturing conformational ensembles that were previously unattainable. The integration of machine learning, exemplified by AlphaFold2, has accelerated structure prediction and dynamics analysis. These approaches have revealed the importance of protein dynamics in allosteric regulation, enzyme catalysis, and intrinsically disordered proteins. The shift towards ensemble representations of protein structures and the application of single-molecule techniques have further enhanced our ability to capture the dynamic nature of proteins. Understanding protein dynamics is essential for elucidating biological mechanisms, designing drugs, and developing novel biocatalysts, marking a significant paradigm shift in structural biology and drug discovery.

## 1. Introduction

Proteins are the fundamental workhorses of cellular processes, playing critical roles in signal transduction, cell division, metabolism, and countless other biological functions [1]. While early structural biology studies portrayed proteins as static entities, it has become increasingly clear that proteins are dynamic molecules undergoing complex motions across multiple timescales [2]. These motions range from femtosecond vibrations of individual atoms to large-scale domain movements occurring over microseconds to milliseconds [3]. Understanding protein dynamics is crucial for elucidating how proteins carry out their diverse functions and how these motions relate to disease states and drug interactions [4].

The field of protein dynamics has seen remarkable progress in recent years, driven by advances in biophysical techniques, computational methods, and the integration of artificial intelligence (AI) approaches [5]. Experimental methods such as nuclear magnetic resonance (NMR) spectroscopy, X-ray crystallography, and cryo-electron microscopy (cryo-EM) have provided unprecedented insights into protein structure and dynamics at atomic resolution [6]. Notably, cryo-EM has emerged as a powerful technique to probe larger-scale dynamics of protein complexes and assemblies, allowing researchers to capture different conformational states and understand how structural rearrangements occur on a broader scale [7,8]. Recent advancements in time-resolved cryo-EM have enabled the capture of transient intermediate states, revealing dynamic processes at near-atomic resolution [9,10]. Similarly, time-resolved X-ray crystallography has been developed to study rapid conformational changes in proteins, offering insights into the atomic details of functional motions occurring on timescales from femtoseconds to milliseconds [11,12,13]. These techniques are revolutionizing our understanding of protein dynamics by allowing the visualization of fleeting states that are crucial for biological function. NMR relaxation experiments have enabled the characterization of protein motions across a wide range of timescales, from picoseconds to seconds [14]. Meanwhile, fluorescence-based techniques, including single-molecule fluorescence resonance energy transfer (smFRET) and high-speed atomic force microscopy (HS-AFM), allow researchers to observe protein motions in real time, revealing rare conformations and heterogeneous behaviors that may be masked in ensemble measurements [15,16,17].

Complementing these experimental approaches, molecular dynamics (MD) simulations have emerged as a powerful tool for studying protein dynamics at atomistic detail [18]. MD simulations can provide a continuous trajectory of protein motions, allowing researchers to observe conformational changes and transient states that may be difficult to capture experimentally [19]. Recent advances in computing power and specialized hardware have enabled simulations to reach biologically relevant timescales of microseconds to milliseconds [18]. The integration of machine learning and AI techniques with traditional biophysical methods has opened up new avenues for studying protein dynamics [6]. Deep-learning models, such as AlphaFold2, have revolutionized protein structure prediction and are now being applied to predict protein dynamics and conformational ensembles [20]. These experimental and computational advances have significantly enhanced our understanding of the biological implications of protein dynamics. One key area is allosteric regulation, where binding at one site affects protein function at a distant site, often involving subtle conformational changes and dynamic processes [21,22].

Advanced NMR techniques and MD simulations have revealed how allosteric signals propagate through protein structures, providing insights into the design of allosteric drugs and the evolution of protein function [23]. Protein dynamics also play a crucial role in enzyme catalysis [24]. While the static lock-and-key model of enzyme–substrate interactions has long been abandoned, recent studies have shown that enzyme dynamics can contribute to catalysis by promoting the formation of reactive conformations, facilitating the sampling of transition states, and modulating the free energy landscape of the reaction [25,26,27]. Understanding these dynamic contributions is essential for rational enzyme design and the development of novel biocatalysts [28].

Furthermore, the study of intrinsically disordered proteins (IDPs) and intrinsically disordered regions (IDRs) has further highlighted the importance of protein dynamics [29]. These proteins and regions lack a stable three-dimensional structure under physiological conditions but play critical roles in cellular signaling and regulation [30]. Advanced NMR techniques, single-molecule fluorescence, and computational methods have revealed how IDPs/IDRs exploit their conformational flexibility to mediate diverse interactions and functions [31]. In the field of structural biology, the recognition of protein dynamics has led to a shift from static structural models to ensemble representations [32]. Ensemble refinement in X-ray crystallography and integrative structural biology approaches are techniques that utilize data from various experimental sources to generate more precise and dynamic models of protein structure and function. These methods aim to better align with experimental data by capturing the different conformations of proteins [33,34,35,36].

Finally, integrating protein dynamics into drug discovery and design has led to new strategies for developing more effective and selective therapeutics [37]. Computational approaches that account for protein flexibility, such as ensemble docking and molecular dynamics-based virtual screening, have improved the ability to identify novel drug candidates and predict their binding modes [38]. Additionally, targeting specific dynamic states or allosteric sites of proteins has emerged as a promising approach for modulating protein function and developing drugs for previously “undruggable” targets [39].

## 2. Experimental Techniques for Studying Protein Dynamics

### 2.1. Innovations in Cryo-EM and X-ray Crystallography for Dynamics Studies

**Figure 1:**
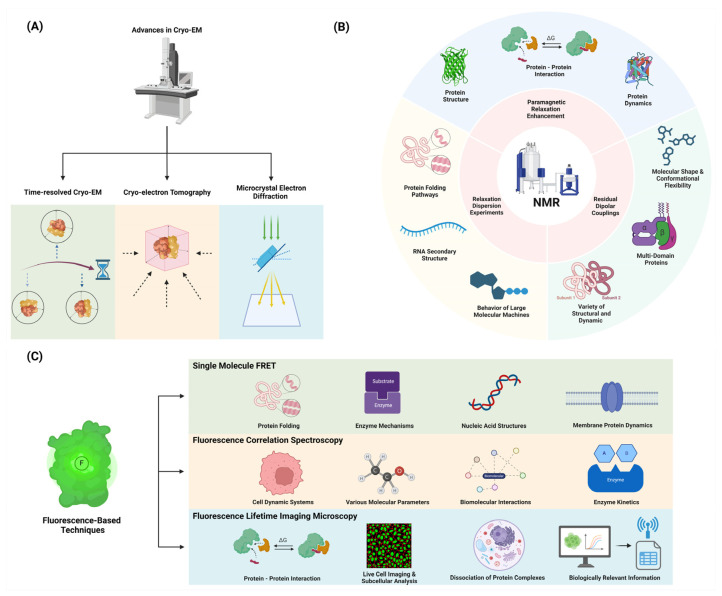
Advanced Experimental Techniques and Applications for Studying Protein Dynamics. (**A**) Schematic diagram of advanced Cryo-Electron Microscopy (Cryo-EM) techniques, including Time-resolved cryo-EM, Cryo-electron tomography, and Micro-crystal electron diffraction (MicroED). (**B**) Application fields of protein dynamics research using Nuclear Magnetic Resonance (NMR) Spectroscopy techniques, including Relaxation dispersion experiments, Paramagnetic relaxation enhancement (PRE), and Residual dipolar couplings (RDCs). These methods are powerful tools for examining structural changes and complex molecular in-teractions of proteins. (**C**) Application fields of protein dynamics research using Fluorescence-Based Techniques, including Single-molecule FRET, Fluorescence correlation spectroscopy (FCS), and Fluorescence lifetime imaging microscopy (FLIM). These methods are utilized as powerful tools to investigate biomolecular dynamics, intracellular protein interactions, and structural changes of molecules, providing high sensitivity and the ability to identify proteins in living cells.

Cryogenic Electron Microscopy (Cryo-EM) has revolutionized structural biology by enabling the visualization of proteins in near-native states without the need for crystallization. Recent developments in cryo-EM have provided unprecedented insights into protein dynamics, particularly for large, flexible complexes (Figure 1).

#### 2.1.1. Time-Resolved Cryo-EM: Capturing Protein Motions at Different Time Points

Time-resolved cryogenic electron microscopy (cryo-EM) is an emerging technique in structural biology that allows researchers to capture structural states that are too transient for standard methods [9]. This technique has revolutionized the field by enabling the visualization of proteins in near-native states without the need for crystallization [40]. Recent developments in cryo-EM have provided unprecedented insights into protein dynamics, particularly for large, flexible complexes [41]. By freezing samples at different time points, time-resolved cryo-EM can trap non-equilibrium states and determine conformations present after defined periods, typically in the millisecond time frame [42]. This approach has been instrumental in elucidating the mechanics of molecular machines such as ribosomes and polymerases, which undergo complex, multistep processes during their functional cycles [41]. Methods such as microsecond time-resolved cryo-EM have enabled observations of protein dynamics, revealing detailed pictures of conformational changes that occur on short timescales [40]. These advancements highlight the potential of time-resolved cryo-EM to fundamentally advance our understanding of protein function and dynamics [9,42].

#### 2.1.2. Cryo-Electron Tomography: Visualizing Proteins in Their Cellular Context

Cryo-electron tomography (Cryo-ET) is a cutting-edge technique that enables the visualization of proteins within their native cellular environments, offering unprecedented insights into their structural organization and interactions within the complex milieu of living cells [43]. This method combines the principles of electron tomography with cryogenic preservation, allowing researchers to capture three-dimensional images of biological samples without the need for crystallization or chemical fixation, thus preserving their native states [44]. The ability to visualize intact cells and tissues while maintaining the spatial relationships between proteins and other cellular components represents a significant advancement over traditional imaging methods [45]. Recent technological advancements in Cryo-ET have significantly improved resolution and data acquisition speeds, facilitating the observation of dynamic processes at molecular scales [46]. Furthermore, tools such as subtomogram averaging have enhanced the high-resolution analysis of large molecular complexes, providing crucial information about protein localization and conformational states within their cellular contexts [47]. As research progresses, Cryo-ET holds immense potential for advancing our understanding of biological systems by enabling the direct observation of proteins functioning in situ [48].

#### 2.1.3. Time-Resolved X-ray Crystallography: Studying Room Temperature and Computational Modeling

Recent advancements in time-resolved X-ray crystallography have significantly enhanced our understanding of protein dynamics, particularly through studies conducted at room temperature and the development of new computational approaches for modeling conformational ensembles. Time-resolved X-ray diffraction methods, such as those leveraging X-ray free-electron lasers (XFELs), allow researchers to capture transient intermediate states, offering insights into the dynamic processes that occur on timescales from femtoseconds to milliseconds [49,50]. These techniques have been instrumental in visualizing rapid conformational changes in proteins, which are crucial for understanding their functional mechanisms [12]. Room-temperature crystallography has emerged as a key approach, providing more physiologically relevant data by avoiding the artifacts introduced by cryogenic cooling [51,52]. This method enhances the detection of low-occupancy conformational states and allosteric sites that are often masked in cryogenic conditions [52]. Alongside these experimental advancements, computational methods such as automated multiconformer model building have been developed to better interpret the ensemble data obtained from these studies [36]. Tools like qFit v3.0 (https://github.com/ExcitedStates/qfit-3.0) facilitate the modeling of protein conformational heterogeneity, improving the accuracy of structural models and providing deeper insights into the relationship between protein dynamics and function [36]. Collectively, these advancements are revolutionizing structural biology by enabling a more comprehensive understanding of the dynamic nature of proteins and their roles in biological processes.

#### 2.1.4. Microcrystal Electron Diffraction (MicroED): Studying Small Protein Dynamics

Microcrystal electron diffraction (MicroED) has emerged as a powerful technique for studying protein dynamics, offering unique advantages in structural biology. This method allows for the determination of high-resolution protein structures from micro- or nanosized crystals, which are significantly smaller than those required for traditional X-ray crystallography [10,53]. MicroED is particularly valuable for proteins that are difficult to crystallize or form only small crystals, such as membrane proteins, which are crucial for drug development [54]. Recent advancements have enabled the capture of conformational dynamics in protein microcrystals on femto- to microsecond timescales, providing insights into transient states that are essential for understanding protein function [55]. The technique’s ability to deliver atomic-resolution structures from minute crystals facilitates the study of challenging targets, including ligand-bound protein complexes, which are pivotal in drug discovery [56]. Moreover, MicroED’s integration with computational modeling allows for the accurate interpretation of conformational ensembles, enhancing our understanding of protein dynamics and their implications in biological processes [10]. As the field continues to evolve, MicroED is poised to complement existing structural biology methods, offering new opportunities for the rapid and detailed analysis of macromolecular structures [10,53].

### 2.2. Nuclear Magnetic Resonance (NMR) Spectroscopy

NMR spectroscopy remains a powerful tool for studying protein dynamics, offering atomic-level resolution and the ability to probe motions across a wide range of timescales. Recent advances include the following.

#### 2.2.1. Relaxation Dispersion Experiments: Detecting and Characterizing Excited States

Relaxation dispersion NMR spectroscopy is a powerful technique used to detect and characterize low-populated, transient excited states of biomolecules by quantifying the broadening of NMR resonance lines due to chemical exchange between ground and excited states [57,58]. This method relies on the exchange between highly populated, NMR-visible ground states and sparsely populated, NMR-invisible excited states, transferring information about magnetic resonance properties such as relaxation parameters, chemical shifts, and residual dipolar couplings from the invisible state to the observable species [58]. The technique provides detailed kinetic and thermodynamic data, enabling the study of structural and dynamic properties of these excited states on the millisecond timescale [59]. Various experiments, including Carr–Purcell–Meiboom–Gill (CPMG) and rotating frame relaxation dispersion (R1ρ) methods, are employed to probe these exchange processes, often requiring isotopic labeling of the macromolecules under study [59,60]. Applications of relaxation dispersion NMR have revealed critical insights into protein-folding pathways, RNA secondary structure dynamics, and the behavior of large molecular machines, offering a high-resolution view of intermediate states that are otherwise challenging to study [61,62]. This approach is complementary to other biophysical techniques and can be performed in the absence of denaturants, making it a versatile tool for studying biomolecular dynamics in native-like conditions [63].

#### 2.2.2. Paramagnetic Relaxation Enhancement (PRE): Probing Long-Range Interactions

Paramagnetic relaxation enhancement (PRE) is a powerful technique in nuclear magnetic resonance (NMR) spectroscopy that utilizes the magnetic dipolar interactions between unpaired electrons in a paramagnetic center and nearby nuclei to increase nuclear relaxation rates [64,65]. This effect is measurable at long distances, making it valuable for probing transient, lowly populated states and long-range interactions in macromolecules. PRE can be applied using intrinsic paramagnetic centers in metalloproteins or by introducing paramagnetic labels through chemical modification [65]. The technique is particularly useful for studying protein dynamics, structure, and interactions, as it can provide information on sparsely populated states and conformational changes that are difficult to detect using other methods [66,67]. PRE measurements typically involve comparing nuclear relaxation rates between paramagnetic and diamagnetic samples, with transverse (Γ2) PRE rates generally providing the most reliable and accurate data [65]. Recent advancements have expanded the application of PRE to include solvent accessibility studies, nanostructure determination in materials, and enhancing temporal resolution in NMR experiments [68,69]. The versatility of PRE has made it an invaluable tool in structural biology, materials science, and other fields where understanding molecular interactions and dynamics is crucial [70].

#### 2.2.3. Residual Dipolar Couplings (RDCs): Characterizing Domain Orientations and Flexibility

Residual dipolar couplings (RDCs) are a valuable tool in nuclear magnetic resonance (NMR) spectroscopy for characterizing the relative orientations and flexibility of molecular domains. RDCs arise when molecules in solution exhibit partial alignment, causing an incomplete averaging of spatially anisotropic dipolar couplings, which provides orientation-dependent restraints that are crucial for structural determination [71]. This partial alignment can be achieved using alignment media such as liquid crystalline phases or stretched polymer gels, which create an anisotropic environment necessary for RDC measurements [72]. RDCs are particularly useful for studying multi-domain proteins, as they provide information on the relative orientation of domains and their dynamic behavior [73]. By measuring the dipolar couplings between NMR-active nuclei, RDCs deliver insights into the global molecular shape and conformational flexibility, which are essential for understanding the functional motions and interactions of biomolecules. The technique has been successfully applied to a variety of structural and dynamic studies [74], including the analysis of protein–substrate interactions and the determination of quaternary structures of oligomers in equilibrium with monomers [75]. Recent advancements in RDC measurement and analysis have further expanded its applications, making it an indispensable tool in structural biology and related fields [74].

### 2.3. Fluorescence-Based Techniques

Fluorescence methods offer high sensitivity and the ability to study proteins in solution or in living cells.

#### 2.3.1. Single-Molecule FRET: Probing Conformational Changes in Individual Molecules

Single-molecule Förster resonance energy transfer (smFRET) is a powerful technique for studying the dynamics and interactions of individual biomolecules with high spatial and temporal resolution [15,76,77]. This method relies on measuring the energy transfer efficiency between donor and acceptor fluorophores attached to specific sites on a molecule of interest [76,78]. smFRET can reveal heterogeneous populations, transient intermediates, and dynamic fluctuations that are often masked in ensemble measurements [77,79]. Recent advances have improved the precision and accuracy of smFRET measurements, with studies reporting distance uncertainties of ±2–5 Å [15,80]. The technique has been successfully applied to investigate protein folding, enzyme mechanisms, nucleic acid structures, and membrane protein dynamics [76,77,78]. Developments in multicolor FRET schemes allow probing of more complex biomolecular systems [77]. Additionally, progress in data analysis methods, including hidden Markov modeling and photon-by-photon approaches, enables extraction of kinetic information on microsecond to millisecond timescales [79]. While challenges remain in achieving higher temporal resolution and applying smFRET in cellular environments, ongoing innovations continue to expand its capabilities for elucidating biomolecular structure and function at the single-molecule level [15,80].

#### 2.3.2. Fluorescence Correlation Spectroscopy (FCS): Analyzing Diffusion and Binding Kinetics

Fluorescence correlation spectroscopy (FCS) is a powerful technique for quantifying molecular dynamics and has been widely applied in diverse fields such as biomedicine, biophysics, and chemistry [81]. By analyzing the time correlation of fluorescence fluctuations induced by molecules diffusing through a focused light, FCS can quantitatively evaluate the concentration, diffusion coefficient, and interactions of molecules both in vitro and in vivo [81,82]. The technique measures the spatial and temporal correlation of individual molecules, providing a bridge between classical ensemble and contemporary single-molecule measurements [82]. Typically implemented on a fluorescence microscope, FCS samples femtoliter volumes, making it especially useful for characterizing small dynamic systems such as biological cells. FCS can investigate various molecular parameters, including diffusion coefficients, chemical rate constants, molecular concentrations, and fluorescence brightness. The method’s sensitivity allows for the analysis of extremely low-concentration biomolecules, with applications ranging from studying diffusion and chemical dynamics to monitoring biomolecular interactions and enzyme kinetics. Recent advancements in FCS include dual-color cross-correlation, multi-focus FCS, and scanning FCS, which enhance its capability to probe complex biological environments and interactions [81,83]. Despite its requirement for high signal-to-noise ratios and long time traces, FCS remains a versatile tool, with ongoing developments aimed at improving its temporal resolution and reducing phototoxic effects on living samples [84].

#### 2.3.3. Fluorescence Lifetime Imaging Microscopy (FLIM): Mapping Protein Interactions in Cells

Fluorescence lifetime imaging microscopy (FLIM) is a powerful technique that measures the time-resolved fluorescence decay of fluorophores to generate contrast in microscopy images, providing information beyond traditional intensity-based imaging [85]. FLIM exploits the characteristic excited-state lifetime of fluorophores, which is sensitive to the local molecular environment, enabling the technique to probe various cellular parameters, such as pH, viscosity, and protein interactions. The method typically employs time-correlated single-photon counting (TCSPC) to measure fluorescence lifetimes with picosecond resolution, allowing for precise quantification of molecular dynamics. FLIM is particularly valuable for studying protein–protein interactions through Förster resonance energy transfer (FRET), where the fluorescence lifetime of a donor fluorophore decreases in the presence of a nearby acceptor, indicating molecular proximity within 1–10 nm [86]. Recent advances in FLIM instrumentation, including the development of fast FLIM techniques and improved spatial resolution, have expanded its capabilities for live-cell imaging and subcellular analysis. Computational developments in FLIM data analysis, such as phasor approaches and machine-learning algorithms, have enhanced the extraction of biologically relevant information from complex lifetime datasets [87]. FLIM has found widespread applications in cell biology, including the study of protein conformational changes, ligand–receptor interactions, and the spatiotemporal dynamics of signaling complexes in living cells [86]. The technique’s ability to discriminate between free and bound states of fluorescently labeled proteins makes it particularly suited for mapping the formation and dissociation of protein complexes in various cellular compartments. As FLIM continues to evolve, its integration with other advanced microscopy techniques and the development of novel fluorescent probes promise to further expand its utility in unraveling the intricate molecular interactions that underlie cellular function [87].

## 3. Computational Approaches to Protein Dynamics

### 3.1. Molecular Dynamics Simulations

**Figure 2:**
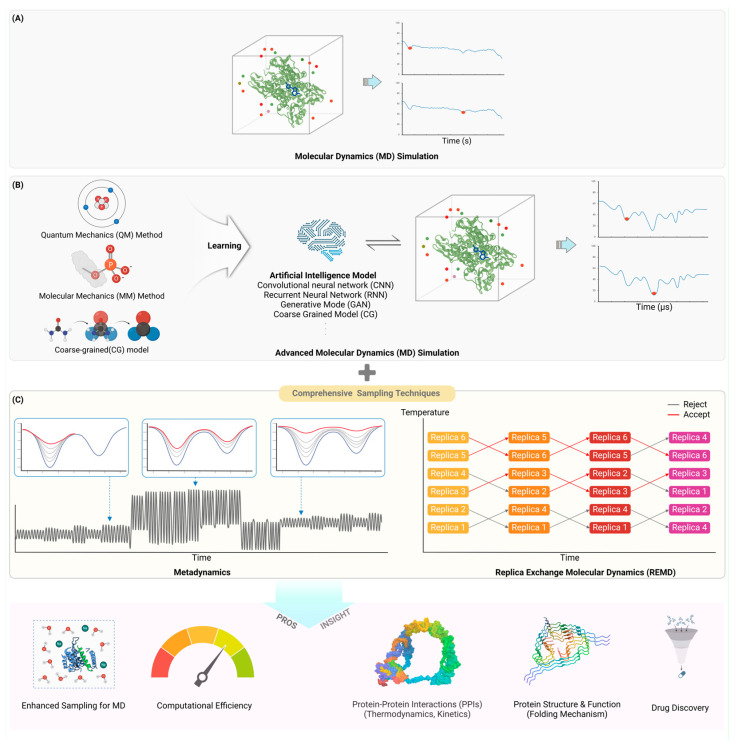
Molecular Dynamics Simulations in Structural Proteomics. (**A**) Molecular dynamics (MD) simulations simulate atomic trajectories over time using Newton’s equations. Capturing atomic behavior throughout time increases temporal efficiency and allows detailed exploration of a large space. MD uses Verlet and Leapfrog algorithms. With the rapid development of computational resources and model development, it outperforms the Verlet and Leapfrog algorithms used in MD. (**B**) Polarizable force fields and quantum mechanics/molecular mechanics (QM/MM) method enable coarse-grained (CG) models to study biological processes on milli-second to second time scales and large-scale protein structural changes. As computing technology has advanced, machine learning (ML) and deep learning (DL) algorithms have showed promise in accuracy and analysis time by learning quantum mechanics data. (**C**) Although various analysis techniques have been developed, force field precision, sampling rare occurrences, and bridging femtosecond to second timeframes remain hurdles. These issues must be resolved to understand biomolecular dynamics and processes. Comprehensive sampling methods like Metadynamics and REMD have emerged to solve these challenges. These improved sampling methods are essential for understanding complicated biological systems and studying structural changes, and they are advancing MD simulation. The merging of ML and DL techniques has made computational resources and MD simulation models essential for understanding new protein dynamics.

Molecular dynamics (MD) simulations have become an indispensable tool for studying protein dynamics at atomic resolution (Figure 2).

#### 3.1.1. Fundamentals of Molecular Dynamics (MD) Simulations: Force Fields and Integration Algorithms

Molecular dynamics (MD) simulations rely heavily on force fields to model the interactions within and between molecules, providing a computational framework for studying molecular systems. A force field is a set of equations and parameters that describe the potential energy of a system, typically decomposed into bonded interactions (including bond stretching, angle bending, and dihedral torsions) and non-bonded interactions (such as van der Waals and electrostatic forces) [88,89]. Bonded interactions are often represented by harmonic potentials, while non-bonded interactions use Lennard–Jones and Coulombic potentials [88,89]. The MD algorithm integrates Newton’s equations of motion to simulate the trajectories of atoms over time, with common integration methods including the Verlet and leapfrog algorithms [36,90]. The Verlet algorithm is favored for its simplicity and time-reversibility, although it does not explicitly calculate velocities, which can be derived separately. The leapfrog algorithm, an alternative, calculates velocities at half-time steps, improving accuracy in velocity calculations. Choosing an appropriate time step is crucial to balance accuracy and computational efficiency; larger time steps increase sampling efficiency but can introduce errors if too large [89,91]. Typically, time steps are chosen to be small enough to accurately capture the fastest motions in the system, such as bond vibrations, while being large enough to efficiently explore the phase space. These principles and methods form the backbone of MD simulations, enabling detailed studies of molecular dynamics across various fields [92,93].

#### 3.1.2. Long-Timescale Simulations: Accessing Biologically Relevant Timescales (ms–s)

Long-timescale molecular dynamics simulations have emerged as a powerful tool for studying biological processes occurring on millisecond to second timescales, overcoming traditional limitations of atomistic simulations [94]. Advances in hardware, including specialized supercomputers and graphics processing units (GPUs), have enabled microsecond to millisecond simulations of protein folding, conformational changes, and ligand binding [94,95]. Enhanced sampling techniques like accelerated molecular dynamics, replica exchange, and Markov state models allow more efficient exploration of conformational space [94,96]. Coarse-graining approaches sacrifice atomic detail but permit much longer effective timescales [97]. The development of polarizable force fields and quantum mechanics/molecular mechanics (QM/MM) methods has improved accuracy for modeling electronic effects [95,97]. Machine-learning potentials trained on quantum mechanical data show promise for combining accuracy and speed [98]. Distributed computing projects like Folding@home have aggregated enormous computational resources for long timescales. Despite these advances, challenges remain in force field accuracy, sampling of rare events, and bridging timescales from femtoseconds to seconds [94,96]. Careful validation against experimental data and assessment of convergence are critical for reliable results [99]. As the field progresses, long-timescale simulations are providing unprecedented insight into the dynamics and mechanisms of biomolecular systems [94,95,98].

#### 3.1.3. Enhanced Sampling Techniques: Exploring Rare Events and Conformational Transitions

Enhanced sampling techniques have significantly advanced molecular dynamics (MD) simulations by enabling the exploration of rare events and conformational transitions, which are crucial for understanding complex biological systems. Metadynamics is a key method that modifies the potential energy landscape by adding a history-dependent bias potential, effectively “filling” free energy wells to facilitate transitions over energy barriers. This approach allows for the detailed exploration of the free energy landscape, as demonstrated in studies of protein folding and ligand binding, where metadynamics has provided insights into the underlying mechanisms of these processes [100,101]. Replica-exchange molecular dynamics (REMD) enhances sampling by simulating multiple replicas of a system at different temperatures, allowing for temperature swaps that help the system overcome energy barriers. This method has been successfully applied to study peptide aggregation and protein folding, revealing detailed conformational landscapes and transition mechanisms [102,103]. Adaptive biasing force (ABF) methods dynamically flatten the free-energy landscape, enabling efficient mapping of complex free-energy surfaces without prior knowledge of the potential energy surface. This approach has been applied to calculate free energy differences in prototypical molecular systems, providing reliable estimates of free energy landscapes [104,105]. Recent advancements also include integrating machine-learning techniques, such as neural networks, to identify collective variables and approximate free-energy surfaces. These methods have improved the efficiency and accuracy of enhanced sampling, as seen in the generation of dynamic protein conformational ensembles, which has enhanced our understanding of protein dynamics and function [106,107]. Collectively, these enhanced sampling techniques expand the capabilities of MD simulations, allowing for the study of processes that occur over extended timescales and providing detailed insights into the dynamic behavior of biomolecules [108].

#### 3.1.4. Coarse-Grained Models: Simulating Large Systems and Complex Assemblies

Coarse-grained (CG) models have become indispensable for simulating large systems and complex assemblies by reducing the number of degrees of freedom, thereby enabling the exploration of larger spatial and temporal scales than all-atom simulations [109,110]. These models achieve computational efficiency by averaging out fast local motions and focusing on slow collective dynamics, which is particularly useful for studying biological processes such as protein folding, membrane dynamics, and large biomolecular complexes [111]. The development of CG models involves defining pseudoatoms that represent groups of atoms and parameterizing the interactions between them, often guided by all-atom simulations or experimental data [112]. Recent advancements in machine learning have further enhanced CG models by improving the mapping from all-atom to CG representations and the accuracy of CG force fields, although there is still a long way to go in achieving fully accurate coarse-gained simulations [109,113]. Techniques such as variational auto-encoders and neural network potentials have been employed to create more accurate and generalizable CG models, capable of capturing essential thermodynamic and kinetic properties of the system. These models have been successfully applied to simulate processes that occur on timescales ranging from microseconds to milliseconds, providing insights into the dynamics of large protein complexes, lipid membranes, and other biomolecular assemblies [110]. Despite their advantages, CG models must be carefully validated against experimental data and all-atom simulations to ensure their reliability and accuracy [111,114]. As the field progresses, the integration of CG models with multiscale simulation approaches promises to bridge the gap between molecular and macroscopic descriptions of biological systems, enhancing our understanding of complex biological phenomena [114,115].

### 3.2. Machine Learning and AI in Protein Dynamics

The integration of machine learning and AI approaches has opened new avenues for understanding protein dynamics.

#### 3.2.1. Deep Learning for Feature Extraction: Identifying Relevant Collective Variables

The integration of machine learning (ML) and AI approaches has opened new avenues for understanding protein dynamics, particularly through deep learning for feature extraction and identifying relevant collective variables (CVs). Deep-learning techniques, such as convolutional neural networks (CNNs) and recurrent neural networks (RNNs), have proven effective in extracting meaningful features from large-scale protein data, enabling the prediction of protein structures and dynamics with improved accuracy [116]. These methods leverage vast protein databases to identify patterns and correlations that are not apparent through traditional approaches, thus enhancing our ability to model protein behavior [117]. One notable application is the use of variational autoencoders and neural network potentials to discover CVs that capture the essential dynamics of protein systems, facilitating the exploration of conformational changes and rare events in molecular simulations [107]. For instance, machine-learning models have been trained on molecular dynamics (MD) simulation data to generate physically realistic conformational ensembles, significantly reducing computational costs and improving the efficiency of sampling techniques [118,119]. This approach has been particularly beneficial for studying intrinsically disordered proteins (IDPs), which exhibit high conformational variability and are challenging to model using conventional methods. By integrating machine learning with MD simulations, researchers can now achieve a more comprehensive understanding of protein-folding mechanisms, energy landscapes, and the thermodynamics and kinetics of protein interactions [117]. As these techniques continue to evolve, they promise to further enhance our ability to predict and manipulate protein functions, with broad implications for drug discovery and protein engineering [116,118].

#### 3.2.2. Generative Models: Predicting Protein Conformations and Dynamics

The integration of generative models in understanding protein dynamics has significantly advanced the prediction of protein conformations and dynamics [120]. Generative models, such as variational autoencoders and generative adversarial networks, have been employed to learn low-dimensional representations of protein conformational spaces, which can then be used to generate novel protein structures and dynamic ensembles [107]. These models are trained on extensive datasets from molecular dynamics (MD) simulations, allowing them to capture the complex energy landscapes and conformational transitions of proteins [120,121]. By learning the underlying probability distributions of protein conformations, generative models can efficiently sample new conformations, thereby reducing the computational cost associated with traditional MD simulations [107]. This approach has been particularly effective in studying intrinsically disordered proteins (IDPs), which exhibit a high degree of conformational variability and are challenging to model using conventional methods [121]. The application of generative models has also facilitated the exploration of rare events and large-scale conformational changes, providing deeper insights into the thermodynamics and kinetics of protein folding and function [120]. As these techniques continue to evolve, they promise to enhance our ability to predict and manipulate protein dynamics, with broad implications for drug discovery and protein engineering [107,122].

#### 3.2.3. Reinforcement Learning: Optimizing Sampling Strategies in MD Simulations

Reinforcement learning (RL) has emerged as a powerful approach for optimizing sampling strategies in molecular dynamics (MD) simulations, enabling more efficient exploration of protein conformational landscapes and rare events [123,124]. RL-based methods, such as REAP (samplingReinforcement Learning Based Adaptive Sampling) and AdaptiveBandit, address the exploration–exploitation dilemma by balancing the sampling of known metastable states with the discovery of new, potentially important regions of the conformational space. These algorithms typically define reward functions that encourage exploration of undersampled areas while also prioritizing regions of interest, such as those with low free energy or specific structural features. For example, FAST (Fluctuation Amplification of Specific Traits) uses a multi-armed bandit-inspired approach to initialize simulations that optimize a given property while maintaining exploration of poorly sampled states [108,125]. Similarly, AdaptiveBandit employs the UCB1 algorithm to find minimum free energy configurations by balancing expected rewards with uncertainty in sampling. Tree search molecular dynamics (TS-MD) applies the upper confidence bounds for trees (UCT) algorithm to efficiently sample transition pathways between initial and target protein configurations [123]. These RL-based methods have demonstrated success in enhancing the sampling of complex systems, such as protein folding and conformational changes, often achieving better coverage of the conformational space compared to traditional MD approaches. Moreover, the integration of RL with other machine-learning techniques, such as deep learning for feature extraction and dimensionality reduction, has further improved the efficiency and accuracy of MD simulations [126]. As the field progresses, RL-optimized sampling strategies promise to play an increasingly important role in unraveling the intricacies of protein dynamics and facilitating drug discovery efforts [123,126].

## 4. Applications and Insights from Protein Dynamics Studies

### 4.1. Protein Folding and Misfolding

**Figure 3:**
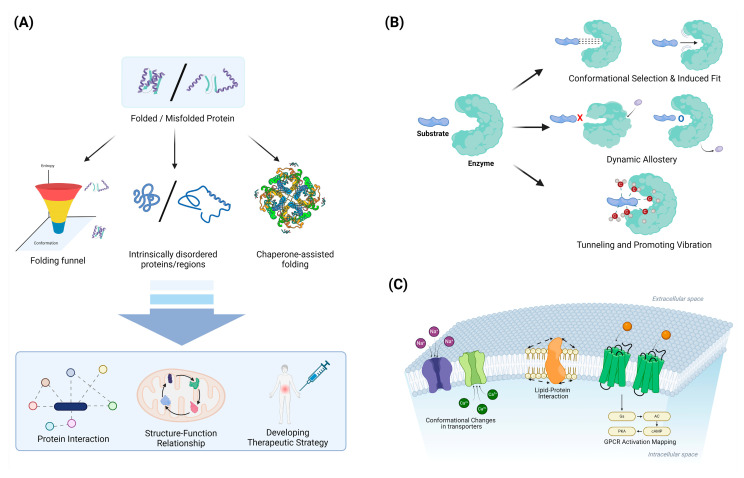
Utilization of Advanced Experimental and Computational Methods in Protein Dynamics Research. (**A**) A schematic diagram illustrating the application of experimental and computational methods to the dynamics of protein folding and misfolding. These methods expedite the identification of targeted protein relationships with drug targets. (**B**) Protein dynamics approaches are employed to apply enzyme-substrate complexes in a schematic diagram. These mechanisms regulate protein activity by utilizing the substrate to undergo conformational and electrochemical changes. (**C**) The application of protein dynamics approaches to membrane proteins. It is capable of predicting the structure of a protein that is bound to a membrane and improving our comprehension of signal transduction.

Combining experimental and computational approaches has led to significant progress in understanding protein-folding mechanisms and the factors contributing to misfolding and aggregation (Figure 3).

#### 4.1.1. Folding Funnels and Energy Landscapes: Characterizing the Thermodynamics and Kinetics of Folding

Folding funnels and energy landscapes provide a powerful framework for understanding the thermodynamics and kinetics of protein folding [127,128,129]. The funnel-shaped energy landscape concept, introduced in the 1990s, revolutionized our understanding of how proteins navigate the vast conformational space to reach their native states [127]. This model depicts folding as a process of descending a rugged energy landscape, where the vertical axis represents free energy and the horizontal axes represent conformational degrees of freedom [128,130]. The overall funnel shape arises from the principle of minimal frustration, which posits that evolution has selected sequences that minimize conflicting interactions, thereby creating a bias towards the native state [129,131]. Quantitatively, the folding funnel can be characterized by parameters such as the energy gap between the native state and the average of misfolded states, the ruggedness of the landscape, and the configurational entropy [128,132]. These parameters influence the folding rate and mechanism, with smoother funnels generally leading to faster folding [130,133]. The presence of local minima in the energy landscape can give rise to folding intermediates and multiple folding pathways, explaining the complexity observed in folding kinetics [132]. Advanced experimental techniques, such as single-molecule spectroscopy and hydrogen exchange mass spectrometry, have provided empirical support for the funnel model by mapping energy landscapes and detecting transient intermediates [131,133]. Computational methods, including molecular dynamics simulations and statistical mechanical models, have further refined our understanding of folding funnels and their relationship to sequence–structure correlations [129,132]. The folding funnel concept has also been extended to explain protein–protein interactions, allostery, and the evolution of new protein functions, demonstrating its broad applicability in structural biology [127,131].

#### 4.1.2. Intrinsically Disordered Proteins: Recognizing the Functional Importance of Structural Flexibility

Intrinsically disordered proteins (IDPs) and intrinsically disordered regions (IDRs) have emerged as crucial components in cellular signaling and regulation, challenging the traditional structure–function paradigm of proteins [29,134]. These proteins lack a stable three-dimensional structure under physiological conditions, instead existing as dynamic ensembles of interconverting conformations [135,136]. The structural flexibility of IDPs/IDRs enables them to interact with multiple binding partners, facilitating their roles as hubs in protein interaction networks and allowing for fine-tuned regulation through post-translational modifications [29]. This conformational plasticity is encoded in their amino acid sequences, which are typically characterized by a low content of bulky hydrophobic residues and a high proportion of polar and charged amino acids [125,136]. IDPs/IDRs are particularly enriched in eukaryotic proteomes and are involved in various cellular processes, including transcriptional control, cell signaling, and the formation of membrane-less organelles through phase separation [29,134,137]. The functional significance of protein disorder extends to enzymes, where IDRs can play important roles in allosteric regulation and catalysis [135,138]. Recent advances in experimental techniques, such as NMR spectroscopy and single-molecule FRET, combined with computational methods, have greatly enhanced our understanding of the structural ensembles and functional mechanisms of IDPs [136,139]. The recognition of the widespread occurrence and functional importance of intrinsic disorder has led to the establishment of the “disorder-function paradigm”, complementing the classical structure–function relationship in protein science [134,140].

#### 4.1.3. Chaperone-Assisted Folding: Elucidating the Role of Cellular Machinery in Protein Folding

Chaperone-assisted protein folding is a critical cellular process that ensures the proper folding and functionality of proteins, preventing misfolding and aggregation, that can lead to cellular dysfunction [141,142]. Molecular chaperones, such as those from the Hsp70 and chaperonin families, play a pivotal role in this process by stabilizing unfolded or partially folded proteins and facilitating their correct folding through ATP-dependent cycles of binding and release [141,143]. These chaperones recognize exposed hydrophobic regions on nascent or stress-denatured proteins, preventing inappropriate interactions that could lead to aggregation [142,144]. For instance, Hsp70 chaperones not only prevent aggregation but also actively remodel misfolded proteins, promoting their refolding into native conformations [143,145]. Additionally, chaperonins like GroEL/GroES provide a protected environment for protein folding, encapsulating substrate proteins and allowing them to fold without interference from the crowded cellular milieu [146,147]. Recent studies using advanced techniques such as single-molecule fluorescence and cryo-electron microscopy have provided detailed insights into the dynamic interactions between chaperones and their substrate proteins, revealing how these molecular machines guide proteins through their folding landscapes [144,148]. The chaperone system is also integral to the cellular stress response, with heat shock proteins being upregulated to manage increased loads of misfolded proteins under stress conditions [142,149]. Understanding the mechanisms of chaperone-assisted folding not only elucidates fundamental aspects of cellular proteostasis but also has significant implications for developing therapeutic strategies against diseases caused by protein misfolding and aggregation [141,147,149].

### 4.2. Enzyme Catalysis and Allostery

Studies of protein dynamics have revealed the critical role of motions in enzyme function and regulation.

#### 4.2.1. Conformational Selection vs. Induced Fit: Understanding Substrate Binding Mechanisms

Conformational selection and induced fit are two fundamental mechanisms that explain how proteins interact with ligands, each offering distinct insights into the dynamics of molecular recognition [150,151]. In conformational selection, proteins exist in a pre-existing ensemble of conformations, and ligand selectively binds to and stabilizes a subset of these conformations, as supported by techniques like NMR and single-molecule FRET [150,152]. Conversely, induced fit posits that the initial protein–ligand interaction triggers a conformational change in the protein to optimize the binding interface, a concept first proposed by Koshland to explain enzymatic specificity [151,153]. These mechanisms are not mutually exclusive and can coexist within the same system, with their relative contributions often dependent on factors such as the ligand concentration and specific protein characteristics [150,154]. Advanced computational methods, including molecular dynamics simulations and enhanced sampling techniques, have been instrumental in elucidating the energy landscapes that govern these binding processes [153,154]. Understanding these mechanisms is crucial for drug design and protein engineering, as it provides insights into how proteins achieve specificity and efficiency in their interactions [150,155]. For instance, in enzyme design, considering both conformational selection and induced fit can lead to more effective catalysts by optimizing both the pre-existing conformational ensemble and the induced changes upon substrate binding [153,154]. In the context of drug discovery, this understanding can guide the development of more potent and selective inhibitors by targeting specific conformational states or by designing ligands that can induce favorable conformational changes [151,154]. Recent studies have also highlighted the role of these mechanisms in allosteric regulation and the function of intrinsically disordered proteins, further expanding their relevance in biological processes [150,154].

#### 4.2.2. Dynamic Allostery: Recognizing the Importance of Entropy in Allosteric Regulation

Dynamic allostery refers to the phenomenon wherein the binding of a ligand leads to conformational changes in a protein that can influence the dynamics of other distant sites within the protein or within associated proteins [156,157]. This process highlights the significance of entropic changes during allosteric regulation, emphasizing that allosteric effects are not merely a consequence of structural changes but also involve shifts in the entropy of the protein states [158]. Entropy plays a crucial role in allosteric regulation as it governs the stability of various protein conformations; thus, changes in entropy upon ligand binding can enhance or inhibit the functional responses of the protein [157]. Additionally, advances in experimental techniques, such as nuclear magnetic resonance (NMR) spectroscopy and molecular dynamics simulations, have demonstrated how conformational flexibility and entropic fluctuations are essential for understanding allosteric mechanisms [158]. This recognition of the importance of entropy in allostery has implications for drug design, providing insights into how small molecules can induce conformational changes that modulate protein activity [159].

#### 4.2.3. Tunneling and Promoting Vibrations: Exploring Quantum Effects in Enzyme Catalysis

Quantum mechanical effects, such as tunneling and promoting vibrations, play a crucial role in enzyme catalysis by influencing the reaction rates and mechanisms involved. Hydrogen tunneling, a process where hydrogen atoms pass through energy barriers rather than over them, has been shown to be significant in enzymatic C–H bond cleavage, linking protein dynamics to catalysis and highlighting the importance of donor–acceptor distance and active-site electrostatics[160]. Promoting vibrations, which are specific localized motions within the enzyme, enhance catalytic efficiency by coupling directly to the reaction coordinate and optimizing the compression along the donor–acceptor distance, thus facilitating hydrogen tunneling[161,162]. These vibrations are believed to be a universal feature of enzymes, contributing to their pre-organized active sites and enabling efficient biochemical transformations. Recent theoretical and experimental studies have demonstrated that these quantum effects are not only significant at cryogenic temperatures but also at physiological conditions, challenging classical transition state theory and emphasizing the dynamic nature of enzyme catalysis[163]. Computational models, particularly quantum mechanical/molecular mechanical (QM/MM) simulations, have provided valuable insights into these mechanisms, underscoring the interplay between electronic structure and protein dynamics in enzyme function[164].

### 4.3. Membrane Protein Dynamics

Advances in studying membrane protein dynamics have provided insights into transport mechanisms and signal transduction.

#### 4.3.1. Lipid–Protein Interactions: Characterizing the Influence of the Membrane Environment

Lipid–protein interactions are fundamental in regulating the structure and function of membrane proteins, influencing processes such as protein conformational changes and membrane domain formation [165,166]. These interactions can be specific, involving direct binding of lipids to proteins, or nonspecific, affecting the general properties of the membrane, such as thickness and lateral pressure [167,168]. Experimental techniques, including cryo-electron microscopy and mass spectrometry, have advanced our understanding of these interactions by providing high-resolution structures and identifying lipid compositions in complex membranes [165,166]. Molecular dynamics (MD) simulations have complemented these experimental approaches, offering detailed insights into the dynamics and energetics of lipid–protein interactions at atomic resolution [168,169]. For instance, MD simulations have revealed how lipid composition and membrane properties modulate the conformational equilibrium of ion channels, such as potassium channels, by altering lateral pressure and membrane thickness [169]. Additionally, specific lipid-binding events have been shown to modulate protein–protein interactions and influence the functional states of membrane proteins, further underscoring the complexity and specificity of lipid–protein interactions [165,167]. Overall, the integration of experimental and computational methods continues to enhance our understanding of the intricate roles that lipid–protein interactions play in cellular membranes [166,168].

#### 4.3.2. Conformational Changes in Transporters: Elucidating Alternating Access Mechanisms

Conformational changes in transporters are essential for elucidating the alternating access mechanisms that facilitate substrate translocation across cell membranes. The alternating access model posits that transporters cycle between outward-facing (OF) and inward-facing (IF) conformations, allowing substrates to bind on one side of the membrane and be released on the other [170,171]. This mechanism has been extensively studied in various transporters, including the Na^+^/Ca^2+^ exchanger NCX_Mj, which demonstrates significant structural asymmetry between its topological repeats, with transmembrane helices TM1–TM6 undergoing substantial displacement during the transition [170]. Crystallographic studies of the sodium-hydantoin transporter Mhp1 have captured the transporter in multiple states, providing a detailed mechanism of how it switches from the OF to the IF state through an occluded intermediate [171]. Molecular dynamics (MD) simulations have further elucidated these transitions, revealing that substrate binding induces conformational changes that facilitate the alternating access mechanism, as observed in SWEET family transporters. Recent advancements in computational approaches, such as AlphaFold2, have enabled the prediction of multiple conformational states of transporters, highlighting the dynamic nature of these proteins and their ability to adopt diverse conformations [172]. The integration of experimental and computational methods continues to enhance our understanding of the alternating access mechanism, underscoring the importance of conformational plasticity in transporter function [173].

#### 4.3.3. GPCR Activation: Mapping the Energy Landscape of Receptor Activation

The activation of G protein-coupled receptors (GPCRs) involves complex conformational changes that can be conceptualized as transitions across an energy landscape [174,175]. This landscape is characterized by multiple energy wells representing distinct conformational states, including inactive, intermediate, and active configurations. Agonist binding modifies the shape of this energy landscape, lowering energy barriers and stabilizing active conformations [174]. The activation process typically involves outward movement of transmembrane helix 6 (TM6), a hallmark of GPCR activation that creates space for G-protein coupling [176]. Studies have revealed a common activation pathway comprising 34 residue pairs and 35 residues that links the ligand-binding pocket to the G-protein coupling region, unifying previously identified key motifs such as CWxP, DRY, and NPxxY. This pathway allows for the decoupling of ligand binding site and G-protein binding region evolution, potentially facilitating the diversification of GPCRs. Advanced techniques like X-ray crystallography, cryo-electron microscopy, and molecular dynamics simulations have been instrumental in mapping these energy landscapes and identifying key conformational intermediates [177,178]. The energy landscape framework provides insights into mechanisms of constitutive activity, inverse agonism, and allosteric modulation, offering a powerful tool for understanding GPCR function and drug design [174,179]. Recent studies have also explored the vibrational energy landscapes of GPCRs, providing graphical representations of residue-level energy distributions in active and inactive states [180].

## 5. Integrating Complementary Techniques for Comprehensive Understanding of Protein Dynamics

Understanding protein dynamics requires a combination of experimental and computational methods, each providing unique insights that, when integrated, offer a more comprehensive view. Experimental techniques such as cryo-electron microscopy (cryo-EM), nuclear magnetic resonance (NMR) spectroscopy, and fluorescence-based methods are powerful for capturing structural and dynamic details at varying resolutions and timescales. Computational approaches, including molecular dynamics (MD) simulations and machine-learning algorithms, offer detailed atomic-level insights and predictive power, complementing experimental observations [181,182].

### 5.1. Cryo-EM and MD Simulations: Synergy in Structural and Dynamic Studies

Cryo-EM has transformed structural biology by enabling the visualization of macromolecular complexes in near-native states without the need for crystallization. Recent advancements, such as time-resolved cryo-EM, allow for capturing snapshots of transient states, providing a static but high-resolution view of dynamic processes. However, these snapshots alone cannot capture the continuous motion of proteins. MD simulations fill this gap by modeling the movements of atoms over time, providing a dynamic picture that complements the static images from cryo-EM. By using cryo-EM-derived structures as starting points for MD simulations, researchers can validate and refine computational models, ensuring that the simulated dynamics correspond to biologically relevant conformations observed experimentally. For example, the conformational changes of ribosomes during translation have been extensively studied using a combination of cryo-EM and MD, revealing the sequence of motions at a level of detail that neither method alone could achieve [181,182].

### 5.2. NMR Spectroscopy and Fluorescence Techniques: Complementary Insights into Protein Dynamics

NMR spectroscopy offers atomic-level resolution for studying protein dynamics, particularly suited for observing conformational changes and interactions over a range of timescales, from picoseconds to seconds. Techniques such as relaxation dispersion and paramagnetic relaxation enhancement (PRE) can detect low-populated, transient states that are often invisible to other structural methods. Meanwhile, fluorescence techniques like single-molecule Förster resonance energy transfer (smFRET) provide real-time data on protein conformational changes and interactions in live cells or under physiologically relevant conditions. When used together, NMR provides detailed structural information about specific states, while smFRET can validate these states and monitor transitions between them in a more dynamic context. For instance, studying the folding pathways of intrinsically disordered proteins (IDPs) benefits greatly from this combination, with NMR identifying the ensemble of conformations and smFRET elucidating the folding kinetics and pathways in real time [183,184].

### 5.3. Computational and Experimental Integration: A Holistic Approach to Protein Dynamics

Molecular dynamics (MD) simulations are invaluable for understanding the movements and flexibility of proteins at atomic resolution, particularly over timescales that are challenging for experimental techniques to probe directly. Enhanced sampling techniques in MD simulations, such as metadynamics and replica exchange, allow researchers to explore rare events and conformational transitions that are critical for understanding biological functions. These simulations can predict how proteins will respond to different conditions or mutations, which can then be experimentally validated. Additionally, machine-learning models have emerged as powerful tools to predict protein conformations and identify key dynamic regions, which can guide the design of experiments using cryo-EM or NMR to test these predictions. This iterative cycle of prediction, experimentation, and validation accelerates the discovery process and deepens our understanding of protein dynamics.

### 5.4. Applications in Drug Discovery and Protein Engineering

The integration of experimental and computational approaches has substantial implications for drug discovery and protein engineering. For example, in the context of drug discovery, understanding the dynamics of G protein-coupled receptors (GPCRs) requires mapping the conformational landscape accessible to these proteins. Combining cryo-EM to capture different receptor states with MD simulations to model the transitions between these states provides a comprehensive view of how potential drugs could stabilize certain conformations. This integrated approach allows for the identification of novel allosteric sites and the design of more effective therapeutics that can modulate protein activity in a controlled manner. Similarly, in protein engineering, combining NMR data on protein flexibility and MD simulations helps design proteins with desired dynamic properties, such as increased stability or enhanced binding affinity.

## 6. Future Directions and Challenges

**Figure 4:**
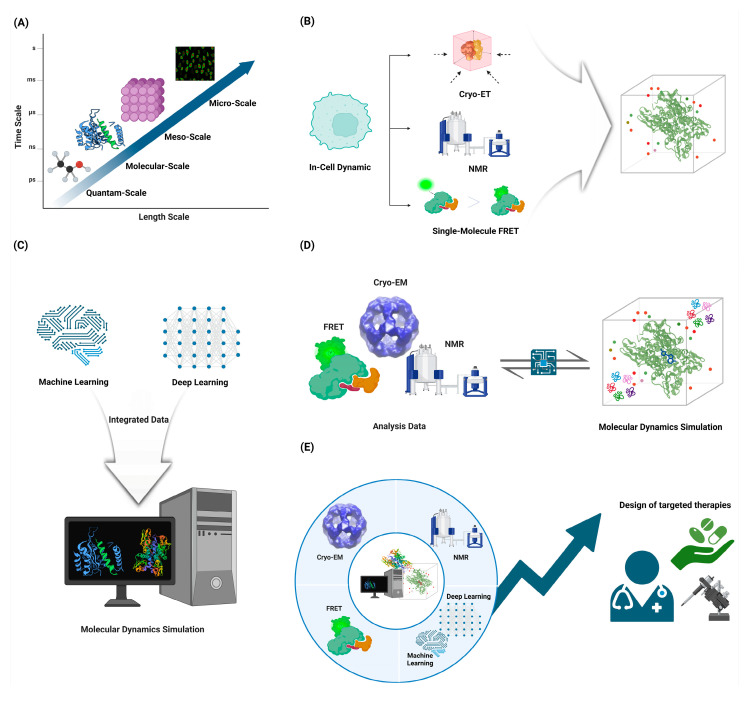
Illustrates the future directions and challenges in protein dynamics research that can be addressed through computational and experimental methods. (**A**) A method for integrating atomic simulations, coarse-grained models, and experimental data to link temporal and spatial dimensions. This multi-scale technique helps understand complex protein behaviours at different resolutions. (**B**) Using advanced imaging and spectroscopic techniques like Cryo-ET, NMR, and single-molecule Fluorescence Resonance Energy Transfer (FRET) to visualize dynamic processes in live cells. (**C**) Forecasting functional protein dynamics and developing tailored proteins using deep learning and machine learning. These AI-driven methods can integrate huge information and rationally design proteins for specific biological purposes and model protein activity more accurately. (**D**) Expanding dynamics studies to massive macromolecular complexes and cellular machinery. By combining MD simulations with Cryo-EM, NMR, and FRET, researchers can gain deep insights into bigger protein assemblies’ dynamic characteristics. (**E**) Linking protein motions to biological functions using cutting-edge technology to simplify targeted therapy. Experimental methods, machine learning, and MD simulations will affect the development of customized medicines to address disease-related protein dysfunctions.

As the field of protein dynamics continues to evolve, several key areas for future research and development emerged (Figure 4).

### 6.1. Limitations of Experimental Techniques for Studying Protein Dynamics

Experimental techniques such as cryo-electron microscopy (cryo-EM), nuclear magnetic resonance (NMR) spectroscopy, and fluorescence-based methods have significantly advanced our understanding of protein dynamics, each with distinct strengths and limitations. Cryo-EM has become a cornerstone in structural biology for studying proteins in near-native states, particularly large, flexible complexes, but it faces challenges in resolving smaller proteins and capturing transient states at a high resolution. Despite improvements like time-resolved cryo-EM, capturing dynamics at millisecond timescales remains difficult due to sample heterogeneity and radiation damage during data acquisition [7,185,186]. Cryo-electron tomography (Cryo-ET) extends the capabilities of cryo-EM by visualizing proteins within their cellular context, providing insights into their organization and interactions in situ, but suffers from lower resolution and longer data acquisition times compared to single-particle cryo-EM [186]. Time-resolved X-ray crystallography and Microcrystal electron diffraction (MicroED) offer high-resolution structural data and can capture transient states but require crystallization, which may introduce artifacts and fail to represent the full range of physiological conditions [12,187]. NMR spectroscopy, particularly through techniques like relaxation dispersion experiments, paramagnetic relaxation enhancement (PRE), and residual dipolar couplings (RDCs), excels in providing atomic-level insights into protein dynamics across a range of timescales but often requires isotopic labeling and faces sensitivity and resolution challenges, especially for large proteins and complexes [184,188]. Fluorescence-based techniques such as single-molecule Förster resonance energy transfer (smFRET), fluorescence correlation spectroscopy (FCS), and fluorescence lifetime imaging microscopy (FLIM) offer real-time insights into protein dynamics in solution or living cells but are limited by photobleaching, fluorophore stability, and the complexity of data interpretation [81,189,190]. Thus, while these experimental techniques have revolutionized our understanding of protein dynamics, each is constrained by specific limitations that affect their applicability to different types of proteins and dynamic processes.

### 6.2. Limitations of Molecular Dynamics Simulations

Molecular dynamics (MD) simulations have become indispensable for studying protein dynamics at atomic resolution, but they are also subject to several critical limitations. One major challenge is the accuracy of force fields, which are the mathematical models used to approximate the forces between atoms; inaccuracies in force field parameters can lead to significant errors in simulated protein conformations and dynamics [91,191]. Moreover, sampling efficiency is a persistent issue in MD simulations, especially for capturing rare events and transitions between different conformational states, which often requires enhanced sampling techniques like metadynamics or replica exchange molecular dynamics (REMD) to overcome high energy barriers [95,191]. The computational cost of MD simulations also remains a significant hurdle, particularly for long-timescale simulations that aim to capture biologically relevant processes occurring on millisecond to second timescales. Although advances in hardware, such as the use of GPUs and specialized supercomputers, have enabled longer simulations, these still require significant resources and often provide limited sampling due to the immense configurational space of proteins [95,192]. Additionally, coarse-grained models, which simplify the representation of molecular systems to extend simulations to longer timescales, often sacrifice atomic detail, leading to potential inaccuracies in describing specific interactions and dynamics critical for understanding protein function [95,193]. Therefore, while MD simulations are powerful tools for exploring protein dynamics, they require careful consideration of their inherent limitations in force field accuracy, sampling efficiency, and computational feasibility.

### 6.3. Integration of Multi-Scale Approaches: Combining Atomistic Simulations with Coarse-Grained Models and Experimental Data to Bridge Timescales and Length Scales

Future directions in computational biology point towards the integration of multi-scale approaches that combine atomistic simulations with coarse-grained models and experimental data to bridge timescales and length scales [194]. Atomistic molecular dynamics simulations provide detailed insights into biomolecular structure and dynamics at the nanoscale but are limited in the timescales they can access [111]. Coarse-grained models allow for simulations of larger systems over longer timescales by reducing the number of degrees of freedom but sacrifice atomic-level detail [195]. Integrating these approaches with experimental data can leverage the strengths of each method while overcoming individual limitations [196]. Machine-learning techniques are emerging as powerful tools to develop accurate coarse-grained models from atomistic simulation data and to bridge between scales [197]. Multiscale modeling frameworks that dynamically couple different levels of theory show promise for capturing processes that span multiple spatiotemporal scales [115]. Key challenges include developing robust methods to systematically connect different scales, quantifying uncertainties across scales, and efficiently sampling rare events [197]. Overcoming these hurdles will enable more comprehensive and predictive models of complex biological systems across scales.

### 6.4. In-Cell Dynamics: Developing Methods to Study Protein Motions in Their Native Cellular Environment

Developing methods to study protein motions in their native cellular environment remains a major challenge and frontier in the field of protein dynamics [198]. While significant progress has been made in studying protein dynamics in vitro, the complex and crowded cellular milieu can significantly alter protein behavior, necessitating in-cell approaches [199]. Recent advances in in-cell NMR spectroscopy have enabled the study of protein structure and dynamics directly within living cells, providing insights into how the cellular environment impacts protein function. Fluorescence-based techniques like single-molecule FRET and tracking of transfected biomolecules are also emerging as powerful tools to probe protein conformational dynamics and interactions in cellular with high spatiotemporal resolution [198]. Cryo-electron tomography combined with computational approaches like tomoDRGN show promise for capturing protein structural heterogeneity and dynamics in their native cellular context [200]. However, significant challenges remain, including improving the sensitivity and resolution of in-cell measurements, developing methods to study low-abundance proteins, and integrating data from multiple techniques to build comprehensive models of protein behavior in cells. Overcoming these hurdles will require continued innovation in labeling strategies, instrumentation, and computational analysis methods [177]. As the field progresses, in-cell dynamic studies have the potential to reveal new principles of protein function and regulation within the complex cellular environment.

### 6.5. AI-Driven Discovery: Leveraging Machine Learning to Predict Functional Motions and Design Proteins with Specific Dynamic Properties

AI-driven discovery is poised to revolutionize our understanding of protein dynamics and enable the design of proteins with specific dynamic properties. Machine-learning approaches have demonstrated remarkable success in predicting protein structures and are now being extended to capture the dynamic nature of proteins [106]. By integrating sequence, structure, and dynamics information, these methods can provide insights into conformational ensembles and functional motions that are crucial for protein function [119]. Deep-learning models, such as those based on geometric deep learning, show promise in predicting protein dynamics from static structures, potentially accelerating the exploration of conformational landscapes [119]. Furthermore, generative models are emerging as powerful tools for designing novel proteins with tailored dynamic properties, opening up new possibilities for protein engineering [201]. The integration of molecular dynamics simulations with machine-learning frameworks allows for more accurate predictions of protein flexibility and allosteric communication pathways. Recent advances in physics-informed neural networks offer the potential to incorporate physical constraints and improve the interpretability of AI-driven predictions in protein science [106]. As these methods continue to evolve, they are expected to play an increasingly important role in understanding protein function at a molecular level and designing proteins with specific dynamic properties for biotechnological and therapeutic applications.

### 6.6. Dynamics in Complex Assemblies: Extending Our Understanding to Large Macromolecular Complexes and Cellular Machines

Future directions in the study of dynamics in complex assemblies focus on extending our understanding to large macromolecular complexes and cellular machines. The dynamic nature of these assemblies significantly impacts their molecular and cellular functions, necessitating advanced methods to study their conformational changes, interactions, and spatial-temporal dynamics within the cell [202]. Recent progress in coarse-grained (CG) modeling has facilitated the study of large biological systems by bridging the gap between microscopic and macroscopic details, allowing for the simulation of complex macromolecular interactions over extended timescales [203]. Techniques such as cryo-electron microscopy (cryo-EM) have revolutionized structural biology by providing high-resolution models of large complexes, enabling the visualization of their dynamic processes during assembly, remodeling, and disassembly [202]. Single-molecule biophysical approaches have also advanced, offering unique insights into the stochastic behavior of individual molecules within these assemblies, thus revealing the non-linear and highly dynamic nature of cellular machinery [204]. However, challenges remain in improving the sensitivity and resolution of these methods to capture the full spectrum of dynamic behaviors in complex environments. Integrating experimental data with computational models, such as all-atom molecular dynamics (MD) simulations and CG models, is crucial for developing comprehensive and predictive models of macromolecular dynamics [203]. As these methods continue to evolve, they promise to enhance our understanding of the intricate dynamics of large macromolecular complexes and their roles in cellular processes [202,203,204].

### 6.7. Linking Dynamics to Function: Developing Quantitative Frameworks to Relate Protein Motions to Biological Function and Disease States

Future directions in linking protein dynamics to function involve developing quantitative frameworks to relate protein motions to biological function and disease states. Proteins are dynamic entities, and their functions are governed by their dynamic personalities, which include fluctuations ranging from atomic vibrations to large domain movements [1]. Understanding these dynamics is crucial, as disease-causing mutations can perturb protein structural networks, leading to altered functions [205]. Integrating experimental data with computational models, such as molecular dynamics (MD) simulations, can reveal how these mutations affect protein stability and function [206]. Techniques like cryo-electron microscopy and NMR spectroscopy have advanced our ability to capture dynamic processes in proteins, providing insights into conformational changes and interactions [1]. Network-based models have also emerged as powerful tools to study the complex spatiotemporal relationships between protein structures and their functions, particularly in the context of disease mutations [207]. These models can help identify allosteric pathways and residues involved in biological activities, linking structural changes to functional outcomes. As we continue to develop and refine these quantitative frameworks, we will enhance our ability to predict how protein dynamics contribute to function and disease, ultimately aiding in the design of targeted therapeutics [208].

## 7. Conclusions

The study of protein dynamics has advanced significantly in recent years, driven by technological innovations in both experimental and computational approaches. As our understanding of protein motions continues to grow, we gain deeper insights into the fundamental mechanisms of life at the molecular level. The integration of diverse techniques, from cryo-EM to AI-powered simulations, promises to further accelerate progress in this field, with far-reaching implications for drug discovery, protein engineering, and our understanding of cellular processes.
